# Association of breathing sound spectra with glottal dimensions in exercise-induced vocal cord dysfunction

**DOI:** 10.1007/s00405-017-4719-0

**Published:** 2017-08-29

**Authors:** Ahmed Geneid, L.-M. Aaltonen, L. Porra, J. Peltonen, K. Palmu, A. Sovijärvi, P. Piirilä

**Affiliations:** 1 Department of Otorhinolaryngology-Head and Neck Surgery, University of Helsinki and Helsinki University Hospital, PL 220, 00029 Helsinki, Finland; 2 Department of Clinical Physiology, HUS Medical Imaging Center, University of Helsinki and Helsinki University Hospital, Helsinki, Finland

**Keywords:** VCD, Vocal cord dysfunction, Stridor, Videolaryngoscopy, Glottal quotient, Paradoxical vocal cord movement

## Abstract

The objective of this study was to evaluate associations between the breathing sound spectra and glottal dimensions in exercise-induced vocal cord dysfunction (EIVCD) during a bicycle ergometry test. Nineteen subjects (mean age 21.8 years and range 13–39 years) with suspected EIVCD were studied. Vocal folds were continuously imaged with videolaryngoscopy and breathing sounds were recorded during the bicycle exercise test. Twelve subjects showed paradoxical movement of the vocal folds during inspiration by the end of the exercise. In seven subjects, no abnormal reactions in vocal folds were found; they served as control subjects. The glottal quotient (interarytenoid distance divided by the anteroposterior glottal distance) was calculated. From the same time period, the tracheal–vocal tract resonance peaks of the breathing sound spectra were analyzed, and stridor sounds were detected and measured. Subjects with EIVCD showed significantly higher resonance peaks during the inspiratory phase compared to the expiratory phase (*p* < 0.014). The glottal quotient decreased significantly in the EIVCD group (*p* < 0.001), but not in the control group. 8 out of 12 EIVCD patients (67%) showed stridor sounds, while none of the controls did. There was a significant inverse correlation between the frequencies of the breathing sound resonance peaks and the glottal quotient. The findings indicate that the typical EIVCD reaction of a paradoxical approximation of the vocal folds during inspiration, measured here as a decrease in the glottal quotient, is significantly associated with an increase in inspiratory resonance peaks. The findings are applicable in the documentation of EIVCD findings using videolaryngoscopy, in addition to giving clinicians tools for EIVCD recognition. However, the study is limited by the small number of subjects.

**Electronic supplementary material:**

The online version of this article (doi:10.1007/s00405-017-4719-0) contains supplementary material, which is available to authorized users.

## Introduction

In vocal cord dysfunction (VCD), a pathological inspiratory approximation of the vocal folds results in dyspnea and the possible generation of stridor sounds. VCD can occur during exercise (exercise-induced VCD, EIVCD), at rest or in both situations, and the etiology is unknown. A number of triggers have been identified, such as physical or psychological stress, respiratory infections, reflux, and indoor air pollutants [1–3]. EIVCD tends to occur among young athletes during hard exercise, with symptoms that resolve spontaneously within seconds to minutes after exercise is terminated. Affected athletes are usually aware of the need to lower their pace when such an episode develops [4, 5].

Among elite athletes, 5% have reported a wheezing inspiratory sound (stridor), with half of those later being diagnosed with asthma [6]. EIVCD can be quite restrictive for athletes who suffer from it, possibly even ending their athletic careers due to the absence of a curative therapy. To our knowledge, few studies have analyzed VCD sounds acoustically. A case study [7] documented the use of lung sound analysis (LSA) for recording a 14-year-old asthmatic girl’s VCD attack; seven microphones were used: six on the chest wall and one over the trachea. The spectrograms of the recorded signals showed continuous monophonic adventitious sounds, attributed to VCD rather than asthma, during both the inspiratory and expiratory phases. The sound was reported to appear first at the anterior neck, followed by the microphones at the lung fields. In another study [8], with a multichannel lung sound analyzer, sounds from the trachea and several locations on the chest were studied, and the stridor sounds of VCD were found to have more symmetrical acoustic characteristics than the peripherally generated wheezing sounds of, e.g., asthma.

Breath sounds in EIVCD have rarely been studied. Acoustic samples collected from several microphones, as reported above, may be problematic in the ambulatory diagnostics of VCD.

EIVCD diagnosis is based mainly on endoscopic findings during the EIVCD attack, with laryngoscopic demonstration of a paradoxical motion of the vocal folds during inspiration being the diagnostic gold standard [9]. In normal breathing, the cross-sectional area between the vocal folds widens, especially during deep inspiration [10], and narrows slightly (<30%) during expiration. The opposite is usually seen in EIVCD attacks or during provocation tests, with a paradoxical adduction of the vocal folds during inspiration.

Previous studies to measure the glottal dimensions in EIVCD are scarce. The diagnostic measurement software was studied by Christensen et al. [11] as a tool in the assessment of EIVCD and exercise-induced laryngomalacia diagnoses. Their study subjects were assessed by continuous laryngoscopic follow-up during an exercise test, and the recorded inspiratory laryngeal images were analyzed by the software, which measured the cross-sectional areas of the glottal aperture. The results showed that exercise-induced laryngomalacia and EIVCD are two separate conditions, both causing laryngeal obstruction, but at different levels. Some studies used quantitative measurements of the abduction and adduction angles, usually by measuring the angle of the vocal fold at the anterior commissure [12].

Objective measurements obtained during clinical endoscopy of glottis patients with suspected EIVCD have been lacking so far. Moreover, depending on the examination center, suspected EIVCD patients may be examined by clinical physiologists and others without visualization of the larynx. Therefore, sound analysis would be helpful for those who do not use flexible endoscopy during the examination. It would also help in the decision making about whether a patient needs an evaluation from an otorhinolaryngologist. Endoscopy shows its value in VCD, and especially EIVCD, as the main glottal change is a reduction of the interarytenoid distance during the paradoxical motion of the vocal folds: the aspect ratio of the interarytenoid distance to the anteroposterior distance can be calculated from an endoscopic picture and this gives rise to a stable quotient.

## Aim of the study

The aim of the study was to assess the acoustic correlates between tracheal sounds and changes in glottal dimensions as detected by fiberoptic videolaryngoscopy during bicycle ergometry in subjects with suspected EIVCD. Glottal dimension measurements, breath sound frequency levels and the occurrence of stridor sounds were compared between subjects with laryngologically diagnosed EIVCD and those with a normal exercise profile to determine the characteristic features of breath sounds during an EIVCD attack.

## Subjects and methods

Nineteen consecutive patients who were referred to the Helsinki University Hospital for suspicion of EIVCD were invited and participated in the study. All of the patients had fiberoptic videolaryngoscopy performed on them during bicycle ergometry. Based on the results, 12 were diagnosed with EIVCD and the seven who were not served as controls. The clinical criterion for EIVCD diagnosis was a paradoxical approximation of the vocal folds and arytenoids during inspiration under videolaryngoscopy. Anthropometric data and medical history information of the subjects were collected (see Table 1). All of the subjects were never-smokers. Three patients with VCD had been diagnosed with asthma earlier, but their asthma medication had stopped and all of them were tested without asthma medication. Two controls had also earlier been diagnosed with asthma, and one of them with occupational methacrylate-induced asthma. Both were tested during asthma medication (peroral montelukast and combination of inhaled budesonide and formoterol fumarate, and the other inhaled beclomethasone). None of the controls or patients had significant (15%) FEV1 reduction after the exercise test, the reduction of FEV1 being less than −10% in all of them, indicating that no asthmatic reaction was found. From those two patients who reported reflux symptoms, only one reported of consuming reflux medications when needed.

**Table 1:** Anthropometric data and medical history of the EIVCD and control groups

	EIVCD group	Control group
Mean age, range	20.5 years (14–39)	24 years (13–36)
Female/male (*n*)	11/1	6/1
Mean height, range	167 cm (155–174)	163.4 cm (157–180)
Mean weight, range	57 kg (45–68)	61 (47–78)
Mean BMI, range	20.4 (17.6–23)	22.7* (19.1–24.8)
Asthma (*n*)^a^	3	2
Allergy to pollens and animals (*n*)	4	2
Reflux (*n*)	2	0

The only significant difference was found for BMI, *p = 0.016

^a^ Number of those with earlier asthma which, however, was not detected in this study

The study had approval from the Ethics Committee of the Department of Surgery, Helsinki and Uusimaa Hospital District. All patients gave their written informed consent.

### EIVCD diagnosis and exercise testing

An ear, nose, and throat examination was performed on all patients. Fiberoptic videolaryngoscopy during bicycle ergometry was performed on all participants according to the procedure utilized by Tervonen et al. [5]. Heart and respiratory rates and a continuous 12-lead ECG follow-up were performed before, during and 5 min after the exercise test, while forced expiratory flow in one second (FEV1) was taken before, immediately after, as well as 4 and 10 min after the test. Blood pressure was measured before and immediately after the test. Oxygen saturation was measured during the test with pulse oximetry from the right earlobe. To avoid irritation caused by the fiberoptic examination, 2% lidocaine on a cotton swab was applied in the patient’s nostril for 5–7 min. The fiberscope used in the examination was held in place by the examining physician. The first workload on the bicycle was usually 40 W for women and 50 W for men, or corresponded to the patient’s reported exercise performance, with an incremental increase of 40 or 50 W, respectively, every 3 min. The exercise was carried out until the patient developed a clinical EIVCD attack with the typical inspiratory paradoxical movement of the vocal folds on videolaryngoscopy or self-reported maximal exertion [i.e., a rating of perceived exertion (RPE) of 19–20 on a scale of 6–20] [13] with a heart rate 85% above their age-expected level.

The videos were reviewed by two otorhinolaryngologists and a clinical physiologist. Diagnosis was based on medical history and identification of the paradoxical movement of the vocal folds during inspiration.

### Acoustic recording and analysis

The acoustic signals were recorded using PulmoTrack^®^ 2020/3020 (a respiratory acoustic monitoring system) by Karmel Sonix, Haifa, Israel [14]. The sensors were coin-shaped piezoelectric elements with a linear 3 dB frequency response of 75–2000 Hz, a resonance at 2.7 kHz, a usable range that extended beyond 4 kHz, and a built-in passive ambient noise rejection capability. The attachment of sensors to the chest wall with adhesive foam pads also reduced ambient noise interference and eliminated contact noise. All sensors were connected to the PulmoTrack, where signal conditioning (amplification 63,000, band-pass filtration 80–4000 Hz at 24 dB/oct) was performed prior to analog-to-digital conversion (11,025 samples/s-1 in Channel 1).

The acoustic sensors were attached as recommended by the manufacturer: one on the right side of the patient’s neck close to the trachea, anterior to the sternocleidomastoid muscle, midway between the thyroid notch (“Adam’s apple”) and the sternal notch (Channel 1), and another on the patient’s right side on the mid-clavicular line at the second intercostal space (Channel 2). Only signals recorded from Channel 1 were utilized in the breath sound analyses due to poor signal quality from Channel 2.

**Fig. 1:**
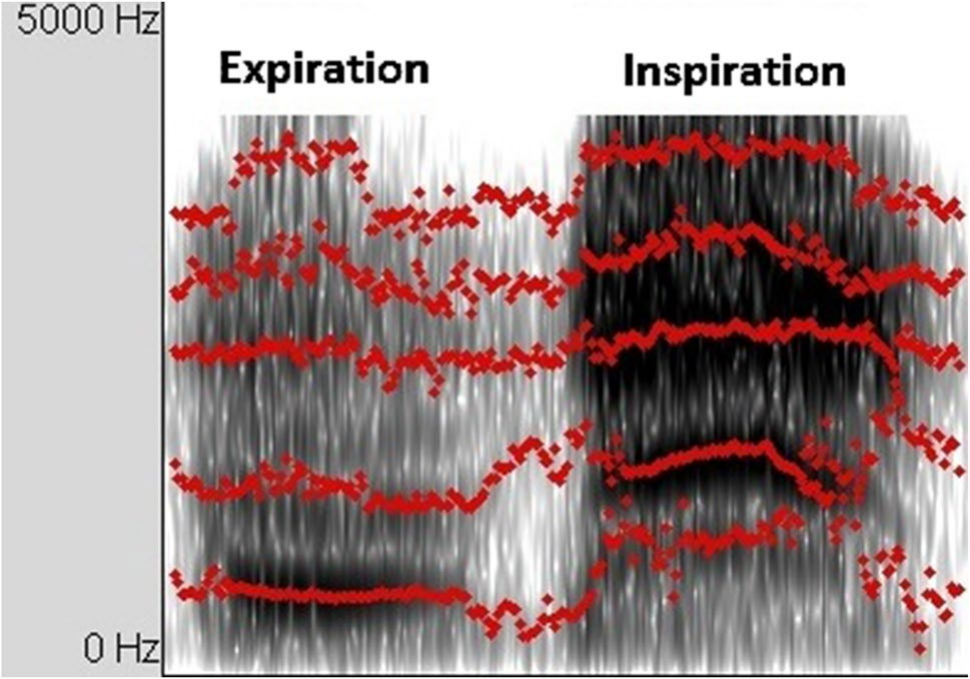
The upper panel shows the audio signal. From left to right: inspiration and expiration. The middle panel shows the resonance peaks as dark bands with obvious higher resonance peaks during the two inspiratory phases. The red lines from below to above represent the different resonance peaks from low to high in Hz

The breath sound analysis was done using the Praat software [15] by extracting the formants of the audio files to define the tracheal–vocal tract resonance peaks in them. In voice analysis, resonance peaks are called formants and defined as the spectral peaks of the sound spectrum of the voice [16]. A formant is a concentration of acoustic energy around a particular frequency and can easily be seen in spectrograms as dark bands. In the present study, where respiratory rather than voice sounds are analyzed, the term formant is, however, rather inaccurate. Accordingly, we refer to such formant-like findings as tracheal–vocal tract resonance peaks (Prof. Johan Sundberg, personal communication, March 31, 2016). Respiratory sounds and their resonance peaks are shown in Fig. 1. As resonance peaks behave like formants, F1, F2, F3, and F4 will be used here to refer to aggregations of resonance peaks increasing in frequency from low to high.

Resonance peaks were extracted from one respiratory cycle during the last 10 s of maximum exertion, immediately before the end of the test. The resonance peaks were obtained for the inspiratory and expiratory phase, as well as the whole cycle.

### Stridor sound detection

**Fig. 2:**
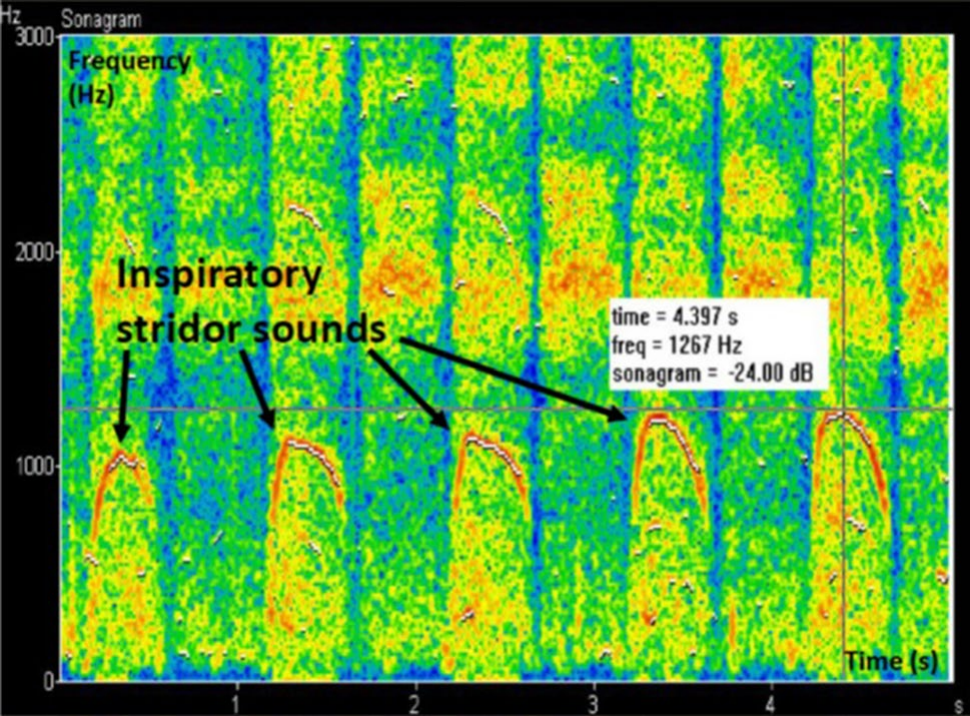
A 20-year-old female who had inspiratory dyspnea, even during moderate exercise. The symptom particularly appeared upon suddenly beginning or rapidly increasing exercise load. Stridor sounds usually accompanied the symptom. The stridor sounds detected by the SuperHelsa analyzer are depicted with arrows. Inspiratory stridor sounds have a fundamental frequency of 1000–1300 Hz; during expiration, no stridor sounds are detected

Stridor sounds were detected and analyzed by the SuperHeLSA lung sound analyzer (Helsinki Lung Sound Analyzer, manufacturer Pulmer Ltd., Helsinki, Finland) [17]. The stridor sounds were detected as continuous monophonic wheezing sounds with sinusoidal waveforms, presented as white lines in the breath sound sonographs (frequency vs. time) (Fig. 2).

The SuperHeLSA lung sound analyzer utilizes a small microphone, a Panasonic WM-60A (Matsushita Electric Industrial Co., Ltd., Kadoma, Osaka, Japan). The microphone has a conical air cavity of 25 mm in diameter and 3 mm deep. The sensitivity of the microphone is 10 mV/Pa and the frequency range in the free field is 20–20 kHz, ±3 dB [17].

### Glottal measurements from videolaryngoscopy

**Fig. 3:**
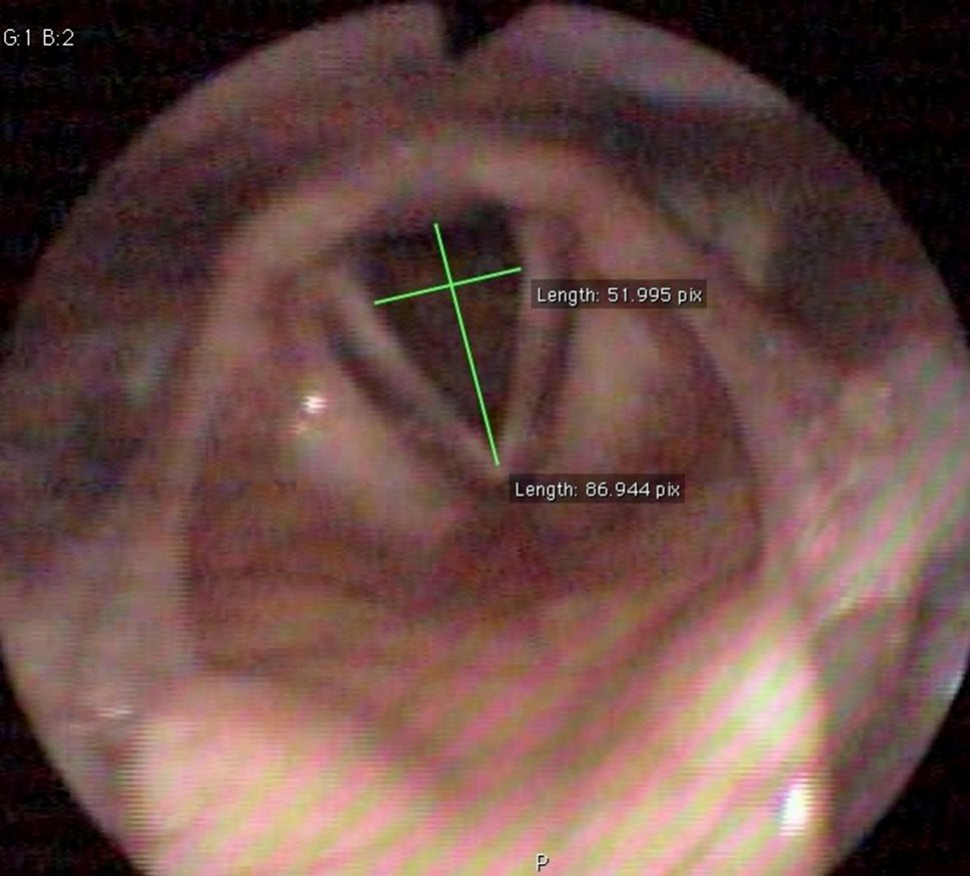
Glottis during maximum expiration of an EIVCD patient

**Fig. 4:**
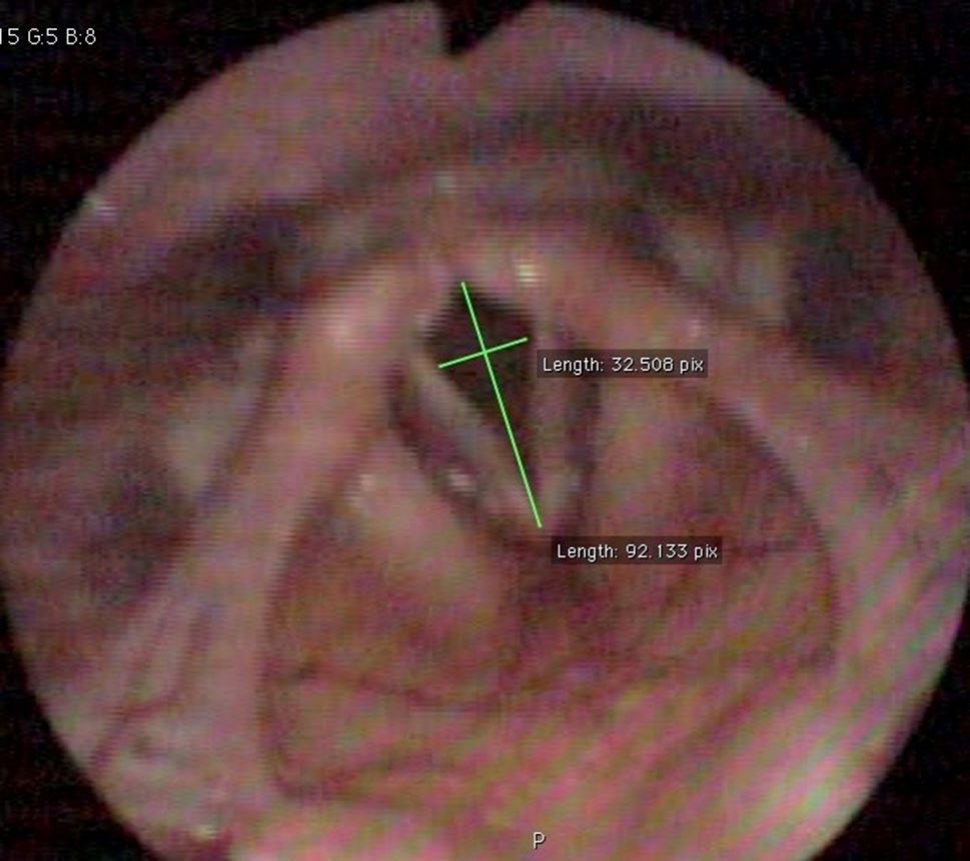
Glottis during maximum inspiration of a EIVCD patient. The photograph shows the diamond-shaped glottis during inspiration, which is typical of EIVCD

To objectively measure the glottal aperture during a VCD attack, we measured the distance, from glottal images, between the vocal processes of the arytenoids, and divided this by the distance, from the endoscopic picture, between the anterior commissure and the midline of the interarytenoid area. We called the generated parameter the glottal quotient. Figures 3, 4 were obtained from an EIVCD patient’s inspiration and expiration phase. The pictures used for calculating the glottal quotient were those corresponding to the recorded respiratory signal; when this was not possible, due to the backward hinging of the epiglottis obscuring the view, another visual cycle was chosen from the same 10 s period at the end of the maximum exertion, immediately before the end of the test.

### Statistics

Statistical analyses were carried out with the SPSS 22 software (IBM SPSS Statistics version 22 for Windows, Armonk, NY, USA). The Mann–Whitney *U* test was used for examining the statistical differences between the EIVCD and control groups in terms of the resonance peak values. The paired samples *t* test was used for analyzing the differences in resonance peak values and glottal quotients between inspiration and expiration, as they were taken from the same subject and respiratory cycle at two timepoints and followed a normal distribution. Normal distribution was validated by the Kolmogorov–Smirnov test. Pearson correlation was used for the correlation measurements.

## Results

Anthropometric measurements showed that the EIVCD group tended to be younger, thinner, and taller than the control group, resulting in a significantly lower mean BMI for the EIVCD group compared to the controls (Table 1).

All subjects had normal laryngeal anatomy and function at rest. In exercise test, none were shown to have asthma based on a reduction of more than 10% in the FEV1 measurements. The level of significant asthmatic reaction would have been a reduction of 15% or more. The mean values of subjective exercise strain were similar (17.2/20 in EIVCD patients and 17.7/20 in controls).

### Acoustic analysis

#### Duration of the respiratory phases

Duration of both the inspiratory and expiratory phases during maximum effort was measured in seconds. EIVCD patients had short inspiration and expiration phases, compared to the control group, which also had longer inspiration than expiration. The inspiratory phase of the EIVCD group was 0.62 ± 0.08 s (mean ± standard deviation), while the control group’s lasted 0.64 ± 0.12 s. The expiratory phase in the EIVCD group was 0.61 ± 0.13 vs. 0.66 ± 0.14 s for the control group. The differences were not significant.

#### Resonance peaks of breath sounds

No difference was found in the resonance peaks of respiratory sounds between males and females. Resonance peaks of the inspiratory and expiratory sounds combined were analyzed. In general, resonance peaks had higher frequencies in the EIVCD group than in the controls. F2, F3, and F4 were significantly higher in the EIVCD group than in the controls (*p* = 0.002, 0.014, and 0.002, respectively). However, F1 was not significantly different upon comparison between the EIVCD and control groups. The mean ± SD resonance peak values for the EIVCD and control groups were, respectively, in Hz, 658 ± 94 and 656 ± 74 for F1; 1650 ± 90 and 1526 ± 52 for F2; 2415 ± 116 and 2286 ± 108 for F3; and 3130 ± 150 and 2926 ± 74 for F4.

Upon comparing the resonance peaks of the inspiratory and expiratory sounds for each group, the inspiratory resonance peaks were significantly higher than the expiratory ones among the EIVCD group for F1, F2, and F3. In addition, F4 was higher during inspiration than expiration, but not significantly higher (Table 2). The control group did not show any significant differences.

**Table 2:** Means and standard deviations (SD) of the resonance peaks of the inspiratory and expiratory sounds for both groups

Group	Resonance peaks	Mean of insp. (Hz)	SD of insp.	Mean of exp. (Hz)	SD of exp.
EIVCD	F1	727*	98	572	95
	F2	1687*	99	1608	119
	F3	2470*	134	2374	136
	F4	3167	176	3112	191
Control	F1	626	26	679	129
	F2	1561	43	1494	99
	F3	2290	124	2313	129
	F4	2907	61	2937	112

*p* values are shown for the comparison between inspiratory and expiratory resonance peaks within each group

*Insp.* inspiratory sound, *Exp.* expiratory sound

* *p* < 0.014

### Stridor analysis by SuperHeLSA lung sound analyzer

Stridor sounds were detected in 8 of the 12 patients with EIVCD, compared to none in the controls. The sounds were monophonic, i.e., one basic stridor frequency with its harmonics, corresponding to the term stridor [18, 19]. The stridor sounds were detected only upon inspiration in five EIVCD patients, and in both inspiratory and expiratory phases in the other three. The mean duration ± standard deviation of inspiratory stridor sounds was 0.282 ± 0.062 s with a mean frequency of 845 ± 129 Hz, while those of expiratory stridor sounds were 0.222 ± 0.09 s and 517 ± 196 Hz, respectively. The four patients who did not have stridor sounds also had higher resonance peaks in the inspiratory phase in comparison to the expiratory, similar to those with stridor sounds.

Examples of a sonograph (frequency vs. time view) from inspiratory stridor sounds in EIVCD are presented in Fig. 2. In addition, two videos from from SuperHeLSA recordings show the findings mentioned above. Video 1 is from the same patient, as shown in Fig. 2, with inspiratory stridor sounds. Video 2 has both inspiratory and expiratory stridors.

### Glottal quotient

The glottal quotients between the inspiratory and expiratory phases of the EIVCD and the control groups were compared. In the EIVCD group, there was a significant difference in the glottal quotient of the inspiratory phase compared to the expiratory one, with the glottal quotient diminishing markedly during inspiration compared to expiration. This difference was not found in the control group (Table 3). The four EIVCD patients without stridor sounds also showed a decrease in glottal quotient during inspiration.

**Table 3:** Means and standard deviations (SD) of the glottal quotient during inspiration and expiration among the EIVCD and control groups

	Phase	Mean	SD
EIVCD	Expiration	0.59	0.18
	Inspiration	0.42*	0.20
Control	Expiration	0.69	0.21
	Inspiration	0.71	0.24

Difference between the inspiratory and expiratory phases within groups was significant for the EIVCD group

* *p* < 0.001

Correlations between the glottal quotient and resonance peaks were studied separately for the inspiratory and expiratory phases, as well as for the whole cycle. The correlation was calculated for both controls and EIVCD patients. The rationale for this was to see if the glottal area reduction, measured by the glottal quotient, is an explanation for the increase in resonance peak values. The glottal quotient of the inspiratory phase had an overall negative correlation with the resonance peaks, measured from both the whole cycle and the inspiratory phase (Table 4). The glottal quotient of the expiratory phase did not show any correlation with the resonance peaks.

**Table 4:** Correlations between the glottal quotient during the inspiratory phase and resonance peaks measured from the whole cycle and the inspiratory phase

Resonance peaks measured from	F1	F2	F3	F4
Whole cycle				
Correlation	−0.368	−0.622**	−0.473*	−0.340
Sig. (2-tailed)	0.121	0.004	0.041	0.155
Inspiratory phase				
Correlation	−0.586**	−0.570*	−0.363	−0.352
Sig. (2-tailed)	0.008	0.011	0.126	0.140

* *p* < 0.05, ** *p* < 0.01

## Discussion

Both the acoustical analysis and the glottal quotient calculations revealed important and significant differences between the EIVCD and control groups. The breath sound resonance peaks of the inspiratory phase were significantly higher than the resonance peaks of the expiratory phase in the EIVCD group. This difference was not found in the control group. Nevertheless, the calculated glottal quotient representing the measured area was significantly reduced among the EIVCD group during the inspiratory phase, compared to the expiratory. This change was not seen among the control group. In addition, a significant negative correlation between the glottal quotient during inspiration and the respective resonance peaks was found.

The present study also offers new information regarding stridor sounds. Stridor sounds heard in VCD or EIVCD have usually been described as inspiratory [7, 19]. In the present study, 5 (42%) of the patients with VCD showed stridor sounds in inspiration, while 3 of 12 (25%) of EIVCD patients showed both inspiratory and expiratory stridor sounds. In addition, we found that 33% of the patients with EIVCD had no stridor sounds. If stridor sounds were present, they appeared at maximum exertion, were monophonic, and disappeared rapidly after the exercise.

In asthma, obstruction is usually peripheral, in contrast to VCD. Asthmatic wheezing sounds are hypothesized to be generated by a fluttering of the airways [20, 21]; when upon sound analysis, several fundamental frequencies seem to join together to generate polyphonic wheezing with a duration of at least 100 ms [19] and a rather varying frequency of 100–5000 Hz [18]. Certain dynamic or fixed obstructions of the airways or larynx, however, generate a loud, high frequency, monophonic stridor sound [18, 22]. Stridor sounds usually occur during inspiration, being generated upon narrowing of the extrathoracic airways or at the laryngeal level. Expiratory stridor sounds have been thought to be generated during intrathoracic obstruction or fixed obstruction of the airways [19]. In a VCD attack, an instantaneous fixed obstruction may be generated at the laryngeal level, explaining the expiratory sound present in some patients in the present study. In the present study, we did not aim to compare asthmatic sounds with VCD sounds. However, three of the patients in the present study had been treated in the past with asthma medication, although no signs of asthma were found in the present examinations. This underlines the importance of differential diagnosis between these disorders, as well as the development of methods for differential diagnosis.

Normal tracheal sounds are said to be generated in the upper airways at the level of the pharynx, glottis, and subglottal region [23] by turbulent airflow and jet formation at the glottis, which causes pressure fluctuations. Airway wall vibrations are also involved in the broad spectrum of the tracheal sound. During maximal exercise, airflow through the trachea and bronchi increases, while, simultaneously, the intensity and frequency of tracheal sounds increase [24, 25]. If there is a narrowing of the trachea or bronchi or a narrowing or closure of the glottal aperture, the airflow increases at a higher rate and the frequency of inspiratory sounds also gets higher than in healthy subjects. This phenomenon was also seen here in the EIVCD patients without stridor sounds.

Studies examining the acoustic correlates of EIVCD or VCD are scarce. Only two earlier studies were found on sound during a VCD attack, and they implemented rather tedious methods, with several microphones on the thoracic wall [7, 8]. In the present study, we used the sound signals from a single microphone, making the measurements easier to collect and more feasible in a clinical setting.

Although endoscopic laryngeal confirmation will probably continue to be the gold standard in VCD and EIVCD diagnosis, measurement of the vocal fold and glottis dimensions during exercise remains difficult. One of the ways to objectively measure the glottis is by laser triangulation [26]. Such a method can usually be applied with rigid endoscopy, in which the laser triangulation device is attached to the endoscope. However, the fiberoptics used in exercise laryngoscopy limits the possibilities of laser triangulation. A study by Kobler et al. in 2006 developed a prototype fiberoptic transnasal laryngeal endoscope with an additional optical system for calibration of images. However, they reported rather high margins of error for lesions with well-defined borders, ranging from 14 to 50% [27].

The glottal quotient, developed in this study, showed a significant reduction in the EIVCD group and an inverse correlation with the resonance peaks of the inspiratory phase. Diagnosis of EIVCD from endoscopical video images is easier with the present method of glottal quotient measurement, compared to some earlier studies [11]. The glottal quotient offers a logical and rather simple way of detecting glottal area changes within a patient. However, before the glottal quotient can be used in diagnosis, normal and abnormal values for the quotient would need to be established and the method of measurement tested on a larger sample.

It is also interesting to find that four of the 12 EIVCD patients did not have stridor sounds detected by the SuperHeLSA lung sound analyzer. Still, acoustic analysis by the Praat software showed that they experienced the same result of higher resonance peaks during inspiration as did the EIVCD patients with stridor sounds. Accordingly, the possibility of screening VCD patients using acoustic analysis is an intriguing area for future research. One of the common diagnostic problems of VCD is that, for some patients, the attacks may occur at rest. For these patients, provocation of an attack using a treadmill or bicycle ergometry is usually unsuccessful.

An important limitation of the study is that it was impossible to blind the photos and videos, as clear approximation would always refer to VCD. Moreover, our clinical physiologist was part of the team that carried out the diagnostic tests on the patients, and unfortunately, it was not possible to have her blinded as well.

This preliminary study, with a small sample size, limits the conclusions that can be drawn based on its findings. A larger sample would help to set the normal values of the glottal quotient.

## Conclusions

This study offers a new diagnostic procedure for assessing patients with suspected VCD or EIVCD by measurement of the glottal quotient, which aids the otorhinolaryngologist in documenting the diagnostic findings from the endoscopic glottal images filmed during exercise. Although EIVCD diagnosis is based on an endoscopic finding of laryngeal inspiratory obstruction, objective measurements for its documentation have been lacking. Measurement of the glottal quotient from endoscopic recordings, developed in the present study, helps in this diagnostic documentation process. The present results show that, among EIVCD patients, the resonance peaks of the inspiratory sounds were significantly higher than the expiratory peaks. They resonance peaks also correlated with the reduction of the glottal quotient. This helps to better understand the pathophysiology of reduced glottal dimensions and the increased frequency of resonance peaks during inspiration. Better knowledge of the high-frequency breath sounds and stridor sounds occurring in VCD can help the clinician to avoid confusion between VCD and asthma diagnoses. Further development of methods for use in respiratory sound analysis might open up new possibilities in EIVCD diagnostics.

## Electronic supplementary material

Below is the link to the electronic supplementary material.

Supplementary material 1 (MOV 6231 kb)

Supplementary material 2 (MOV 5591 kb)
